# Identification and statistical optimization of fermentation conditions for a newly isolated extracellular cholesterol oxidase-producing *Streptomyces cavourensis* strain NEAE-42

**DOI:** 10.1186/s12866-016-0830-4

**Published:** 2016-09-20

**Authors:** Noura El-Ahmady El-Naggar, Nancy M. El-Shweihy, Sara M. El-Ewasy

**Affiliations:** Department of Bioprocess Development, Genetic Engineering and Biotechnology Research Institute, City of Scientific Research and Technological Applications, New Borg El-Arab City, Alexandria, 21934 Egypt

**Keywords:** Cholesterol oxidase, *Streptomyces cavourensis* strain NEAE-42, Plackett-Burman design, Central composite design, Identification, 16S rRNA

## Abstract

**Background:**

Due to broad range of clinical and industrial applications of cholesterol oxidase, isolation and screening of bacterial strains producing extracellular form of cholesterol oxidase is of great importance.

**Results:**

One hundred and thirty actinomycete isolates were screened for their cholesterol oxidase activity. Among them, a potential culture, strain NEAE-42 is displayed the highest extracellular cholesterol oxidase activity. It was selected and identified as *Streptomyces cavourensis* strain NEAE-42. The optimization of different process parameters for cholesterol oxidase production by *Streptomyces cavourensis* strain NEAE-42 using Plackett–Burman experimental design and response surface methodology was carried out. Fifteen variables were screened using Plackett–Burman experimental design. Cholesterol, initial pH and (NH_4_)_2_SO_4_ were the most significant positive independent variables affecting cholesterol oxidase production. Central composite design was chosen to elucidate the optimal concentrations of the selected process variables on cholesterol oxidase production. It was found that, cholesterol oxidase production by *Streptomyces cavourensis* strain NEAE-42 after optimization process was 20.521U/mL which is higher than result obtained from the basal medium before screening process using Plackett-Burman (3.31 U/mL) with a fold of increase 6.19.

**Conclusions:**

The cholesterol oxidase level production obtained in this study (20.521U/mL) by the statistical method is higher than many of the reported values.

## Background

Cholesterol oxidase (3ß-hydroxysteroid oxidase, EC 1.1.3.6) is a flavin adenine dinucleotide (FAD)-dependent enzyme belongs to the family of oxidoreductases, specifically those acting on the CH-OH group of donor with oxygen as acceptor that in most cases catalyzes the oxidation and isomerization of cholesterol (cholest-5-en-3β-ol) using oxygen as an electron acceptor to form 4-cholesten-3-one (cholestenone) and hydrogen peroxide [1].

Cholesterol oxidase enzyme is simple, specific, and furthermore profoundly sensitive; utilized for the clinical determination of cholesterol levels in serum, HDL, or LDL for the evaluation of atherosclerotic illnesses and different lipid problems and also for assessing the danger of thrombosis [2]. A high blood cholesterol level is regarded to be related to cardiovascular disorder and its degradation products (cholesterol oxides) have been hypothesized to be associated with colon cancer [3]. Therefore, it has been proposed that bacterial degradation of cholesterol in cholesterol containing foods might also be beneficial for human health [4]. In addition, cholesterol oxidase has been used for the bioconversion of a variety of sterols and non-steroidal compounds, allylic alcohols [5]. Moreover, cholesterol oxidase has potential applications as a biocatalyst which can be used as an insecticide that is a necessary part of pest manages strategies using transgenic crops [6]. Cholesterol oxidase is additionally implicated in the manifestation of some of the bacterial diseases (tuberculosis), viral (HIV) and non-viral prion origin (Alzheimer’s) [7]. In current years, a variety of electrochemical biosensors using the immobilized cholesterol oxidase have been pronounced for the determination of cholesterol in serum or food [8]. Since the assays using this enzyme are simple, specific, and distinctly sensitive compared with the traditional chemical methods, its use has emerged widespread. Cholesterol oxidases can additionally be used to produce a precursor for chemical synthesis of steroid hormones [1] and to degrade of dietary cholesterol in foods [4].

Cholesterol oxidase is an extracellular or an intracellular enzyme produced by many microorganisms. Cholesterol oxidases may additionally be intrinsic membrane bound enzymes located on the outside of the cell or can be recovered from broth filtrate as an extracellular enzyme. Cholesterol oxidases have been recovered from different microorganisms such as *Nocardia* [9], *Streptomyces* [10] and *Streptoverticillium* [11]. Cholesterol-assimilating bacteria produce a cholesterol oxidase, which is involved in the first step of cholesterol metabolism [12]. Some pathogenic bacteria possess cholesterol oxidases, which are a major membrane-damaging factor and consequently implicated in the pathogenicity of these bacteria [13].

The objective of this study is to obtain an efficient cholesterol oxidase producer for industrial and medicinal needs, to optimize culture conditions using response surface methodology for high production of cholesterol oxidase by *Streptomyces cavourensis* strain NEAE-42.

## Methods

### Microorganisms and cultural conditions

*Streptomyces* spp*.* used in this study are local isolates isolated from various soil samples collected from different localities of Egypt and kindly provided by Dr. Noura El-Ahmady El-Naggar (Department of Bioprocess Development, Genetic Engineering and Biotechnology Research Institute, City of Scientific Research and Technological Applications, Alexandria, Egypt). These isolates were maintained on slopes containing starch-nitrate agar medium of the following composition (g/L): Starch 20; KNO_3_ 2; K_2_HPO_4_ 1; MgSO_4_.7H_2_O 0.5; NaCl 0.5; CaCO_3_ 3; FeSO_4_.7H_2_O 0.01; agar 20 and distilled water up to 1 L. The isolates were stored as spore suspensions in 20 % (v/v) glycerol at −20 °C for subsequent investigation.

### Qualitative screening for cholesterol oxidase producing microorganisms using colony staining method

Cholesterol oxidase is the enzyme which able to convert cholesterol into hydrogen peroxide and cholest-4-en-3-one. Medium consists of (g/L): Cholesterol 2, KNO_3_ 2, K_2_HPO_4_ 1, MgSO_4_.7H_2_O 0.5, NaCl 0.5, CaCO_3_ 3, FeSO_4_.7H_2_O 0.01, agar 20 and distilled water up to 1 L was used for plate screening. Agar plates were seeded with spores of actinomycetes and incubated at 30 °C for 7 days. Cholesterol oxidase producing potentialities was performed on the grown colonies using colony staining method. Discs of filter papers were dipped into the solution containing 0.5 % cholesterol; 1.7 % 4-aminoantipyrine; 6 % phenol and 3000U/l horseradish peroxidase in 100 mM potassium buffer phosphate (pH 7.0). Thereafter, soaked discs had been located on grown colonies on the plates and incubated at room temperature for 24 h. Development of pink color in the medium surrounding the tested colonies due to the quinoneimine dye formation is due to the activity of cholesterol oxidase [14]. The strain which showed the most promising result was selected for further investigations.

### Inoculum preparation

250 mL Erlenmeyer flasks containing 100 mL of broth medium containing: glucose 12 g/L; starch 9 g/L; yeast extract 6 g/L; peptone 4 g/L; (NH_4_)_2_SO_4_ 7.5 g/L; cholesterol 2 g/L; K_2_HPO_4_ 1 g/L; MgSO_4_.7H_2_O 0.5 g/L; FeSO_4_.7H_2_O 0.02 g/L; NaCl 1 g/L; MnSO_4_ 0.008 g/L; CaSO_4_ 0.002 g/L; ZnSO_4_ 0.002 g/L; CaCl_2_ 0.0002 g/L; Tween 80 0.05 g/L [15] were inoculated with 9 mm diameter five disks taken from the 7 days old stock culture grown on starch nitrate agar medium. The inoculated flasks were incubated for 48 h in a rotatory shaker incubator at 30 °C and 200 rpm and were used as inoculum for subsequent experiments.

### Production conditions

100 mL of fermentation medium were dispensed in 250 mL Erlenmeyer conical flasks, inoculated with the previously prepared inoculum. The inoculated flasks were incubated on a rotatory shaker incubator at 150–200 rpm and 30–37 °C. After the specified incubation time for each set of experimental trials, the mycelium of the isolate was collected by centrifugation at 6000 rpm for 15 min. The cell free supernatant was used for assay of the enzyme activity.

### Assay of enzyme activity

Cholesterol oxidase activity was measured by hydrogen peroxide estimation generated during cholesterol oxidation process. In this reaction, hydrogen peroxide was coupled with 4-aminoantipyrine and phenol by peroxides to produce quinoneimine dye with maximum absorption at 500 nm. Cholesterol dissolved in Triton X-100 (non-ionic detergent) was used as substrate for the reaction. The reaction mixture was consisted of 3 μmol of cholesterol in 1.0 mL of 1 % Triton X-100, 300 μmol of phosphate buffer, pH 7.0, 0.1 mL of enzyme solution, 21 μmol of phenol and 20 U of horseradish peroxidase, 1.2 μmol of 4-aminoantipyrine in a final volume of 3 mL. Reaction was incubated at 37 °C for 10 min with shaking. This reaction was terminated by heating at 100 °C for 3 min. One enzyme unit was defined as the amount of enzyme that librated 1 μmol of H_2_O_2_ per minute at 37 °C.

### Morphology and cultural characteristics of the selected strain

The spore chain morphology and the spore surface ornamentation of strain NEAE- 42 were examined on inorganic salt/starch agar (ISP medium 4) after incubation for14 days at 30 °C using the coverslip technique of Kawato and Shinobu [16]. The dehydrated, gold-coated specimen can be examined with Analytical Scanning Electron Microscope Jeol JSM-6360 LA operating at 20 Kv at different magnifications at the Central Laboratory, City of Scientific Research and Technological Applications, Alexandria, Egypt. Cultural characteristics were observed on ISP media 1–7 according to the methods described by Shirling and Gottlieb [17]; all plates were incubated at 30 °C for 14 days.

### Chemotaxonomy and physiological characteristics

The whole-cell sugars were identified by high performance liquid chromatography analysis. Carbon source utilization was tested on plates containing ISP basal medium 9 and melanoid pigment production was examined following the methods of Shirling and Gottlieb [17] on ISP media 1, 6 and 7. Sodium chloride tolerance was determined according to the methods of Tresner et al. [18]. Casein degradation was evaluated following the method of Gordon et al. [19] and reduction of nitrates to nitrites was examined [20]. Liquefaction of gelatin was determined by using the method of Waksman [21]. The ability for coagulation or peptonization of milk was evaluated as described by Cowan and Steel [22]. According to the method of Nitsch and Kützner [23], Lecithinase activity was conducted on egg–yolk medium and the capacity to decompose cellulose was tested following the method of Ariffin et al. [24]. The strain ability to produce α-amylase was determined [25]. The antimicrobial activity of the organism was tested against four bacterial (*Staphylococcus aureus*, *Pseudomonas aeruginosa*, *Klebsiella* and *Escherichia coli*), and five fungal strains (*Alternaria solani*, *Rhizoctonia solani*, *Fusarium oxysporum*, *Bipolaris oryzae* and *Fusarium solani*).

### 16S rRNA sequencing

The preparation of genomic DNA of the strain was conducted in accordance with the methods described by Sambrook et al. [26]. The PCR amplification reaction was performed according to the methods described by El-Naggar et al. [27]. Sequencing was performed and sequencing product was deposited in the GenBank database under accession number KJ676478.

### Sequence alignment and phylogenetic analysis

The 16S rRNA gene sequence of strain NEAE- 42 was aligned with the corresponding 16S rRNA sequences of the type strains of representative members of the genus *Streptomyces* retrieved from the GenBank, DDBJ, EMBL and PDB databases by using BLAST program (https://blast.ncbi.nlm.nih.gov/Blast.cgi?PAGE_TYPE=BlastSearch) [28]. Multiple alignment and phylogenetic tree analysis was performed using the software package MEGA4 version 2.1 [29], the tree was constructed using the neighbour-joining method of Saitou and Nei [30]. The phylogenetic tree, sequence data and alignments used to produce the results displayed in Fig. 3 have been deposited in TreeBASE (https://treebase.org/treebase-web/home.html).

### Screening of main factors influences cholesterol oxidase production by Plackett–Burman design

In order to determine the variables affecting cholesterol oxidase production, different nutritional and environmental variables were evaluated in a two-steps experimental design strategy. In the first step, a two factorial design, Plackett–Burman statistical experimental design, is used to identify the critical variables which required for elevation of cholesterol oxidase production. Plackett–Burman statistical experimental design is very useful for screening the most important factors for enzyme production with respect to their main effects [31, 32]. The total number of the experiments which must be carried out according to Plackett–Burman design is *n* + 1, where *n* is the number of variables [33]. A total of 15 independent (assigned) and four unassigned variables (which commonly referred as dummy variables) were screened in Plackett–Burman experimental design. Dummy variables (D_1_-D_4_) are used to estimate experimental errors in data analysis. The fifteen different independent variables are shown in Table 1 including cholesterol, starch, glucose, yeast extract, peptone, (NH_4_)_2_SO_4_, K_2_HPO_4_, NaCl, MgSO_4,_ FeSO_4,_ temperature, incubation time, inoculum size, agitation speed and pH were chosen to be screened by Plackett Burman experiment. Each variable is represented at two levels, high and low denoted by (+) and (−), respectively. The experiment in 20 runs was conducted to study the selected variables effect on cholesterol oxidase production. All trials were performed in duplicate and the average of cholesterol oxidase activities were used as responses. Plackett–Burman experimental design is based on the first order model:

1 $$ Y={\beta}_0+{\displaystyle \sum {\beta}_i{X}_i} $$

Where, Y is cholesterol oxidase activity (response or dependent variable), β_0_ is the model intercept and *β*_*i*_ is the linear coefficient, and X_i_ is the level of the independent variable.

**Table 1:** Experimental independent variables at two levels used for the production of cholesterol oxidase by *Streptomyces cavourensis* strain NEAE-42 using Plackett–Burman design

Code	Variables	Levels	
		−1	+1
A	Temperature (°C)	30	37
B	Incubation time (days)	5	7
C	Inoculum size (%, v/v)	2	4
D	Agitation speed (rpm)	150	200
E	pH	7	8.5
F	Glucose (g/L)	10	15
G	Starch (g/L)	7	10
H	Cholesterol (g/L)	1	2
J	Yeast extract (g/L)	4	6
K	Peptone (g/L)	3	5
L	Ammonium sulphate (g/L)	6	8
M	K_2_HPO_4_ (g/L)	0.5	1
N	NaCl (g/L)	0.5	1
O	MgSO_4_ (g/L)	0.2	0.5
P	FeSO_4_ (g/L)	0.0	0.02

### Optimization of cholesterol oxidase production by response surface methodology

The central composite design (CCD) under the response surface methodology (RSM) was used to elucidate the optimal values of the most significant independent variables. In this study, the experimental plan consisted of 20 runs and the independent variables were studied at five different levels (−1.68, −1, 0, 1, 1.68). All the runs were performed in duplicate and the average of obtained cholesterol oxidase activity was taken as the dependent variable or response (Y). The experimental results were fitted to the following second order polynomial model:

2 $$ Y={\beta}_0+{\displaystyle \sum_i{\beta}_i{X}_i+{\displaystyle \sum_{ii}{\beta}_{ii}}{X_i}^2+}{\displaystyle \sum_{ij}{\beta}_{ij}{X}_i{X}_j} $$

In which Y is the predicted response, β_0_ is the regression coefficients, β_i_ is the linear coefficient, β_ii_ is the quadratic coefficients, β_ij_ is the interaction coefficients, X_i_ and X_j_ are coded levels of independent variables.

### Statistical analysis

Design Expert® 7.0 software version 7 (Stat-Ease Inc., USA) for Windows was used for the experimental designs and statistical analysis. The statistical software package, STATISTICA software (Version 8.0, StatSoft Inc., Tulsa, USA) was used to plot the three-dimensional surface plots, in order to illustrate the relationship between the responses and the experimental levels of each of the variables utilized in this study.

## Results and Discussion

The total of one hundred and thirty morphologically different actinomycete strains were qualitative screened for their cholesterol oxidase activity using colony staining method, formation of pink zones around the colonies indicated the presence of cholesterol oxidase activity. The strain which showed the large pink zone around the colony was selected for further experiments. The selected strain was cultured in100 mL of fermentation medium and the cell free supernatant was used for enzyme activity assay. The most promising isolate was selected and identified based on morphological, cultural, physiological and chemotaxonomic properties, as well as 16S rRNA sequence.

### Morphological and cultural characteristics

**Fig. 1:**
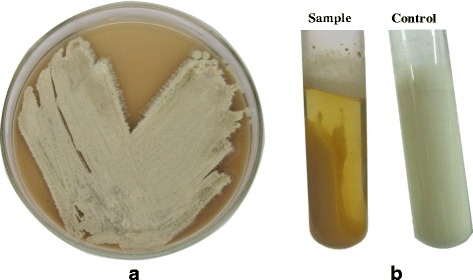
**a** Color of the aerial mycelium of *Streptomyces* sp. NEAE-42 grown on starch-nitrate agar medium for 7–14 days of incubation at 30 °C, **b** coaggulation and peptonization of milk

Cultural properties of strain NEAE-42 are shown in Table 2. Aerial mass color is white to olive beige on yeast extract-malt extract agar (ISP medium 2) and olive green on starch-nitrate agar medium (Fig. 1a). Strain NEAE-42 grew well on ISP medium 2–7. The color of the substrate mycelium was not sensitive to pH changes. The mycelium does not fragment and the verticils are not present. Diffusible pigments were faint brown on most test media.

**Table 2:** Culture characteristics of the *Streptomyces* sp. strain NEAE-42

Medium	Color of			Growth
	Aerial mycelium	Substrate mycelium	Diffusible pigment	
ISP medium 2 (Yeast extract -malt extract agar)	White to olive beige	Brown	Faint brown	Excellent
ISP medium 3 (Oatmeal agar)	Olive green	Olive brown	Faint brown	Excellent
ISP medium 4 (Inorganic salt-starch agar)	Olive green	Olive brown	Faint brown	Excellent
ISP medium 5 (Glycerol asparagines agar)	Olive beige	Brown	Faint brown	Excellent
ISP medium 6 (Peptone-yeast extract iron agar)	Faint beige	Faint yellowish brown	Non-pigmented	Excellent
ISP medium 7 (Tyrosine agar)	Faint beige	Faint brown	Non-pigmented	Excellent

The substrate mycelium pigment was not pH sensitive when tested with 0.05 N NaOH or 0.05 N HCl

The diffusible pigment was pH sensitive when tested with 0.05 N NaOH or 0.05 N HCl, yellow in acidic, brown in alkaline

**Fig. 2:**
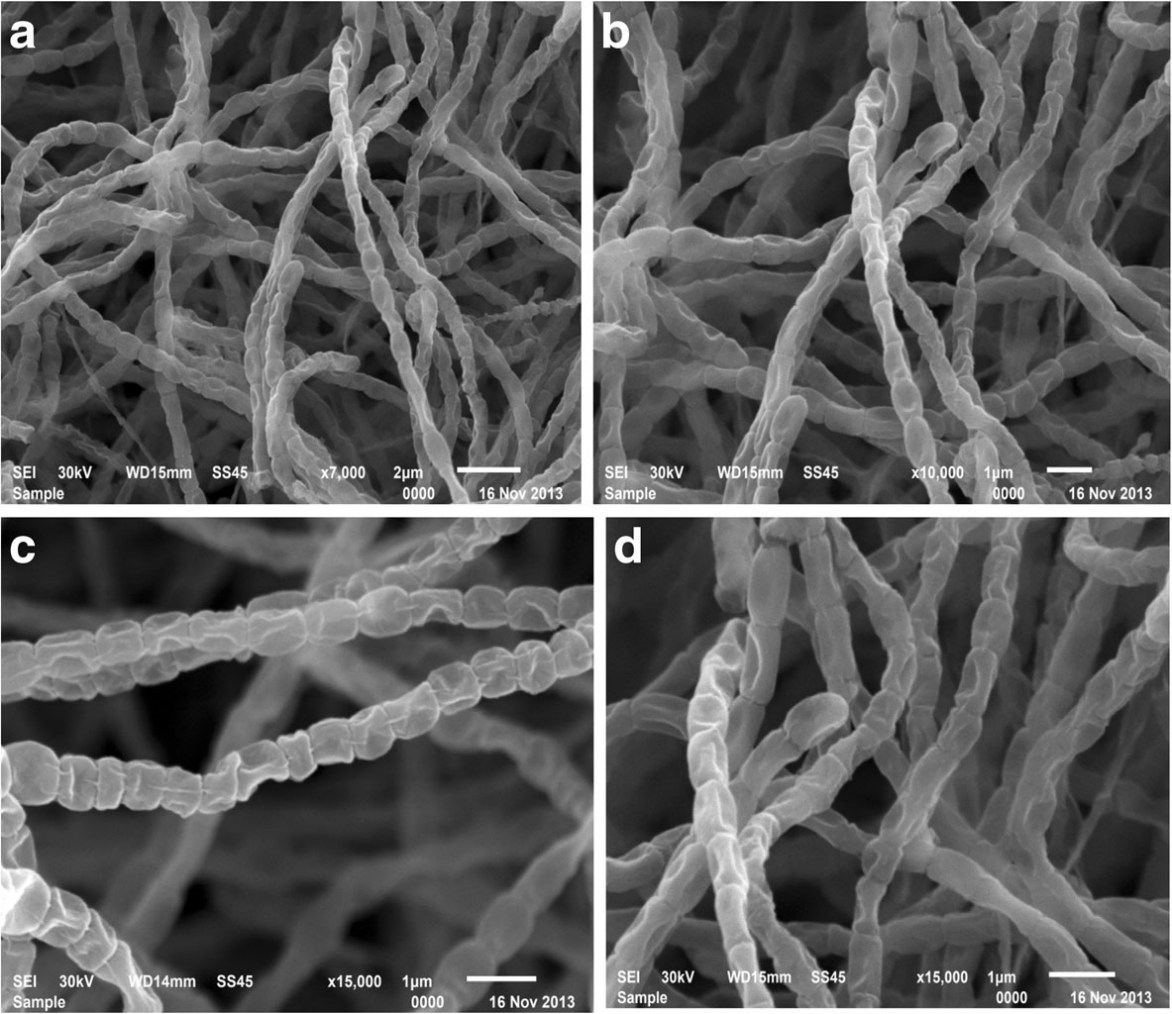
Scanning electron micrograph showing the spore-chain morphology and spore-surface ornamentation of strain NEAE −42 grown on starch nitrate agar medium for 14 days at 30 °C at magnification of 7000 X (**a**), 10000 X (**b**) and 15000 X (**c**, **d**)

The scanning electron micrograph of strain NEAE-42 which cultured on starch nitrate agar medium revealed that the organism produced an extensively branched substrate mycelium and aerial hyphae which differentiated into *Rectiflexibiles* type spore-chains carrying more than 50 elongated, irregular and smooth-surfaced spores (Fig. 2).

### Chemotaxonomy and physiological characteristics

The physiological and biochemical characteristics of strain NEAE-42 are shown in Table 3. The strain produced faint brown diffusible pigments on most test media. Production of melanin was positive on peptone-yeast extract iron agar medium. Starch hydrolysis, casein hydrolysis, milk coagulation and peptonization (Fig. 2b) and nitrate reduction were positive. Gelatin liquification and lecithin degradation were negative. α–amylase (starch hydrolysis), cellulase (growth on cellulose), protease (degradation of casein), chitosanase, uricase and asparaginase of strain NEAE-42 were produced while lecithinase, and gelatinase were not produced. Strain NEAE-42 has no antimicrobial activities against *Staphylococcus aureus, Bacillus subtilis, Escherichia coli, Pseudomonas aeruginosa, Klebsiella pneumonia, Alternaria solani, Bipolaris oryzae, Rhizoctonia solani, Fusarium oxysporum, Aspergillus niger,* and *Candida albicans.* D (−) fructose, D (+) galactose, D (+) xylose, L-arabinose, D (+) glucose, ribose, sucrose, D (+) mannose, cellulose and maltose are utilized for growth but trehalose is not utilized for growth. No growth or only traces of growth with rhamnose and raffinose. The whole-cell hydrolysates contained mainly xylose and galactose.

**Table 3:** Phenotypic properties that separate strain *Streptomyces* NEAE-42 from related *Streptomyces* species. Data for reference species were taken mainly from Bergey’s Manual^®^ of Systematic Bacteriology -volume five the actinobacteria [35]

Characteristic	*Streptomyces* sp. strain NEAE-42	*Streptomyces cavourensis*	*Streptomyces flavolimosus*	*Streptomyces badius*	*Streptomyces flavogriseus*	*Streptomyces fimicarius*	*Streptomyces microflavus*
Aerial mass color on ISP mdeium 2	White to olive beige	Yellow or white	White or pale yellow	Yellow or intermediate between gray and yellow	Gray	Yellow or white	Gray or yellow color
Reverse side of colony on ISP medium 2	Brown	Moderate to strong brown		No distinctive pigments	Strong yellow or orange-yellow	Orange or reddish brown	No distinctive pigments
Production of diffusible pigment	Faint brown	Brown	Yellow to brown	Not formed	No pigment, or only a trace of yellow	Red	No pigment formed
Spore chain morphology	Rectiflexibles	Rectiflexibiles	Rectiflexibiles	Rectiflexibiles	Rectiflexibiles	Rectiflexibiles	Rectiflexibiles
Spore surface	Smooth with surface irregularities	Smooth, sometimes with minor surface irregularities	Smooth	Smooth	Smooth	Smooth	Smooth
Spore shape	Rectangular		Oval-shaped				
Sensitivity of diffusible pigment to pH	Yellow in acidic, brown in alkaline					changing to yellow in acidic	
Melanin production on							
Peptone-yeast extract iron agar	+	+	–	–	–	–	–
Tyrosine agar	–	–	–	±	–	–	–
Tryptone-yeast extract broth	–	+	–	±	–	–	–
Maximum NaCl tolerance (%, w/v)	7	7.5		5			2.5
Degradation of							
Lecithin	–						
Casein	+						
Starch	+		+				
Coagulation of milk	+		–				
Peptonization of milk	+		–				
Nitrate reduction	+		+				
Gelatin liquification	–		–				
Utilization of carbon sources (1 %,w/v)							
D(-) fructose	+	+	+	+	+	+	+
D(+) xylose	+	+	–	+	+	+	+
D(+) galactose	+		–				
D(+) glucose	+	+	+	+	+	+	+
L-arabinose	+	+	+	+	+	+	±
Ribose	+						
D(+) mannose	+	+		–	+	+	+
Sucrose	+	±	–	±	–	±	±
Maltose	+						
Rhamnose	±	±	–	±	+	+	+
Raffinose	±	±		±	–	±	±
Cellulose	+		–	–	+	+	
Trehalose	–						

The optimal growth temperature was 30 ^o^C and optimal pH was 7.0. Strain NEAE-42 has no antimicrobial activities against *Staphylococcus aureus, Bacillus subtilis, Escherichia coli, Pseudomonas aeruginosa, Klebsiella pneumonia, Alternaria solani, Bipolaris oryzae, Rhizoctonia solani, Fusarium oxysporum, Aspergillus niger,* and *Candida albicans.* α–amylase (starch hydrolysis), protease (degradation of casein), cellulase (growth on cellulose), uricase, chitosanase and asparaginase of strain NEAE-42 were produced while lecithinase, and gelatinase were not produced

*Abbreviations*: *+* Positive, – Negative, ± Doubtful, *Blank cells* no data available

On the basis of cultural, morphological and chemotaxonomic properties above, as well as the physiological properties of strain NEAE-42 shown in Table 3, it is evident that strain NEAE-42 belongs to the genus *Streptomyces* [34]*.*

### 16S rRNA gene sequence comparisons and phylogenetic analysis

**Fig. 3:**
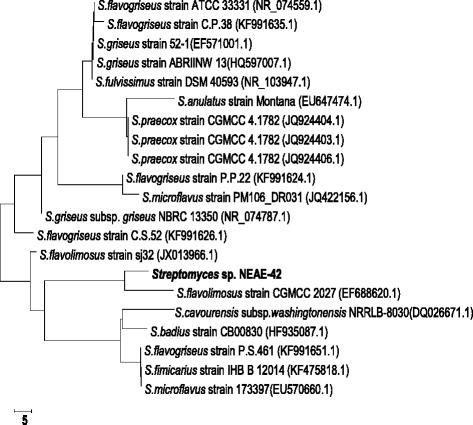
Neighbour-joining phylogenetic tree based on 16S rRNA gene sequences, showing the relationships between strain NEAE-42 and related species of the genus *Streptomyces*. Bar, 5 substitution per nucleotide position

The strain NEAE-42 16S rRNA gene sequence was deposited in the GenBank database under the accession number KJ676478. The complete (1510 bp) sequence of strain NEAE-42 was aligned with the sequences of the genus *Streptomyces* members retrieved from the GenBank databases by using BLAST [28]. The phylogenetic tree (Fig. 3) showed that the isolate falls into one distinct clade with Streptomyces flavolimosus strain CGMCC 2027 (GenBank accession no. EF688620.1), *Streptomyces cavourensis* subsp. *washingtonensis* NRRLB-8030 (GenBank accession no. DQ026671.1), *Streptomyces badius* strain CB00830 (GenBank accession no. HF935087.1), *Streptomyces flavogriseus* strain P.S.461 (GenBank accession no. KF991651.1), *Streptomyces fimicarius* strain IHB B 12014 (GenBank accession no. KF475818.1) and *Streptomyces microflavus* strain 173397(GenBank accession no. EU570660.1) with which it shared 16S rRNA gene sequence maximum identity of 99.0 %.

Based on the collected data and the comparative study of the isolate No. NEAE-42 (Table 3) in relation to the recorded properties of closest related species of the genus *Streptomyces*, it is most closely related to the type strains of *Streptomyces cavourensis* subsp. *washingtonensis* NRRLB-8030 (GenBank accession no. DQ026671.1) (99 % sequence similarity) [35]. Therefore, this strain was identified as *Streptomyces cavourensis* strain NEAE-42.

### Statistical screening of fermentation process variables affecting cholesterol oxidase production by *Streptomyces cavourensis* strain NEAE-42 using two-level fractional factorial design, Plackett-Burman design

Plackett-Burman (PB) design was used to determine which variables significantly affect cholesterol oxidase production by *Streptomyces cavourensis* strain NEAE-42. Compared with other medium design strategies, the Plackett-Burman design is simple, fast method suitable for screening multiple variables in one experiment and is often used to evaluate the most important and significant variables affecting culture requirements for fermentation and enzyme production. In order to evaluate the effect of physical parameters (temperature, incubation time, inoculum size, agitation speed, pH), carbon sources (cholesterol, starch, glucose), nitrogen sources (yeast extract, peptone, (NH_4_)_2_SO_4_), in addition to energy sources (K_2_HPO_4_), and metals (NaCl, MgSO_4_.7H_2_O, FeSO_4_. 7H_2_O) for the maximum production of cholesterol oxidase by *Streptomyces cavourensis* strain NEAE-42, Plackett-Burman design was applied to determine the most important factors.

The Plackett-Burman design for the nineteen variables along with the corresponding responses for cholesterol oxidase production are shown in Table 4. Plackett-Burman experiments showed wide variation (0.000 to 6.910 U/mL) in cholesterol oxidase production; this variation reflected the importance of medium optimization to attain higher cholesterol oxidase production.

**Table 4:** Twenty-trial Plackett–Burman experimental design for evaluation of independent variables with coded values along with the observed cholesterol oxidase activity

Std	Run no.	Coded levels of independent variables																			Cholesterol oxidase activity (U/mL)		Residuals
		A	B	C	D	E	F	G	H	J	K	L	M	N	O	P	Dummy 1	Dummy 2	Dummy 3	Dummy 4	Actual value	Predicted value	
15	1	1	1	1	−1	1	−1	1	−1	−1	−1	−1	1	1	−1	1	1	−1	−1	1	0.397	0.297	0.100
6	2	−1	−1	1	1	−1	1	1	−1	−1	1	1	1	1	−1	1	−1	1	−1	−1	1.522	1.622	−0.100
4	3	1	1	−1	1	1	−1	−1	1	1	1	1	−1	1	−1	1	−1	−1	−1	−1	6.910	6.907	0.003
3	4	1	−1	1	1	−1	−1	1	1	1	1	−1	1	−1	1	−1	−1	−1	−1	1	1.267	1.263	0.003
7	5	−1	−1	−1	1	1	−1	1	1	−1	−1	1	1	1	1	−1	1	−1	1	−1	6.000	5.900	0.100
17	6	−1	1	1	1	1	−1	1	−1	1	−1	−1	−1	−1	1	1	−1	1	1	−1	1.080	1.180	−0.100
18	7	−1	−1	1	1	1	1	−1	1	−1	1	−1	−1	−1	−1	1	1	−1	1	1	2.325	2.225	0.100
8	8	−1	−1	−1	−1	1	1	−1	1	1	−1	−1	1	1	1	1	−1	1	−1	1	1.953	2.053	−0.100
14	9	1	1	−1	1	−1	1	−1	−1	−1	−1	1	1	−1	1	1	−1	−1	1	1	2.011	2.008	0.003
19	10	1	−1	−1	1	1	1	1	−1	1	−1	1	−1	−1	−1	−1	1	1	−1	1	2.260	2.263	−0.003
20	11	−1	−1	−1	−1	−1	−1	−1	−1	−1	−1	−1	−1	−1	−1	−1	−1	−1	−1	−1	0.690	0.687	0.003
2	12	−1	1	1	−1	−1	1	1	1	1	−1	1	−1	1	−1	−1	−1	−1	1	1	0.490	0.487	0.003
9	13	1	−1	−1	−1	−1	1	1	−1	1	1	−1	−1	1	1	1	1	−1	1	−1	0.200	0.100	0.100
16	14	1	1	1	1	−1	1	−1	1	−1	−1	−1	−1	1	1	−1	1	1	−1	−1	0.000	0.003	−0.003
10	15	−1	1	−1	−1	−1	−1	1	1	−1	1	1	−1	−1	1	1	1	1	−1	1	6.600	6.603	−0.003
12	16	−1	1	−1	1	−1	−1	−1	−1	1	1	−1	1	1	−1	−1	1	1	1	1	1.667	1.670	−0.003
1	17	1	1	−1	−1	1	1	1	1	−1	1	−1	1	−1	−1	−1	−1	1	1	−1	4.787	4.887	−0.100
13	18	1	−1	1	−1	1	−1	−1	−1	−1	1	1	−1	1	1	−1	−1	1	1	1	2.076	2.176	−0.100
5	19	−1	1	1	−1	1	1	−1	−1	1	1	1	1	−1	1	−1	1	−1	−1	−1	3.497	3.397	0.100
11	20	1	−1	1	−1	−1	−1	−1	1	1	−1	1	1	−1	−1	1	1	1	1	−1	0.807	0.810	−0.003

The “-1” sign correspond to the minimum value and the “ + 1” sign correspond to the maximum value of the input parameter range

**Fig. 4:**
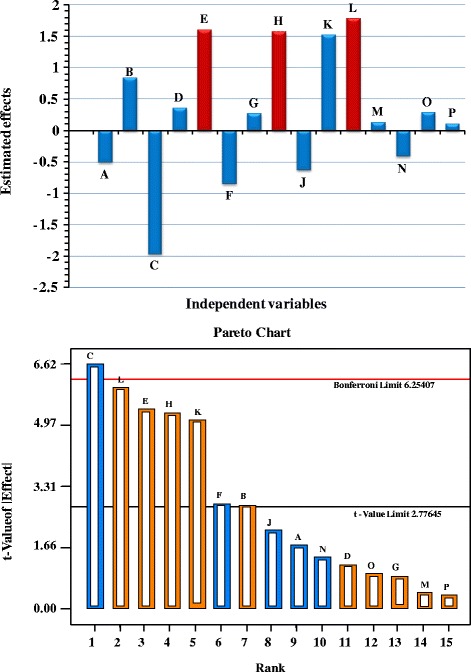
**a** Effect of independent variables on cholesterol oxidase production by *Streptomyces cavourensis* strain NEAE-42 using Plackett-Burman design (The red color represent the most significant positive independent variables affecting enzyme production); **b** Pareto chart illustrates the order and significance of the variables affecting cholesterol oxidase production by *Streptomyces cavourensis* strain NEAE-42 using Plackett-Burman design (the blue colors represent negative effects and the orange color represent positive effects)

Statistical analysis by multiple-regression model of cholesterol oxidase activities was performed which is represented in Tables 5, 6. Table 5 and Fig. 4a shows the main effect of each variable on the cholesterol oxidase production. With respect to the main effect of each variable, we can see that ten variables from the fifteen different independent variables named cholesterol, starch, glucose, peptone, (NH_4_)_2_SO_4_, K_2_HPO_4_, MgSO_4_, FeSO_4,_ incubation time and agitation speed affect positively cholesterol oxidase production, where the other five variables named yeast extract, NaCl, temperature, inoculum size and pH affect negatively cholesterol oxidase production. The variables with positive effect were fixed at high level and the variables which exerted a negative effect on cholesterol oxidase production were maintained at low level for further optimization by a central composite design.

**Table 5:** Regression coefficients, estimated effect and % of contribution for cholesterol oxidase production by *Streptomyces cavourensis* strain NEAE-42 using Plackett-Burman design

Term	Coefficient	Effect	% Contribution
Intercept	2.327	4.654	
A	−0.256	−0.511	3.991
B	0.417	0.834	6.513
C	−0.981	−1.962	15.323
D	0.177	0.355	2.770
E	0.802	1.603	12.521
F	−0.422	−0.845	6.598
G	0.133	0.267	2.083
H	0.787	1.574	12.293
J	−0.314	−0.628	4.903
K	0.758	1.516	11.842
L	0.890	1.781	13.909
M	0.064	0.128	0.997
N	−0.205	−0.411	3.209
O	0.141	0.283	2.210
P	0.054	0.107	0.838

**Table 6:** Regression statistics and analysis of variance (ANOVA) for the experimental results of Plackett-Burman design used for cholesterol oxidase production by *Streptomyces cavourensis* strain NEAE-42

Source	*SS*	*MS*	*F-*value	*t -*Stat	*P*-value Prob > F	Confidence Level (%)
Model	86.172	5.069	101.190	15.705	0.0098*	99.02
A	1.306	1.306	26.067	−1.724	0.0363*	96.37
B	3.476	3.476	69.398	2.814	0.0141*	98.59
C	19.244	19.244	384.161	−6.620	0.0026*	99.74
D	0.629	0.629	12.553	1.196	0.0713	92.87
E	12.849	12.849	256.492	5.410	0.0039*	99.61
F	3.568	3.568	71.219	−2.851	0.0138*	98.62
G	0.356	0.356	7.100	0.900	0.1167	88.33
H	12.386	12.386	247.254	5.311	0.0040*	99.6
J	1.970	1.970	39.336	−2.118	0.0245*	97.55
K	11.494	11.494	229.447	5.117	0.0043*	99.57
L	15.856	15.856	316.522	6.009	0.0031*	99.69
M	0.081	0.081	1.626	0.431	0.3304	66.96
N	0.844	0.844	16.847	−1.387	0.0545	94.55
O	0.400	0.400	7.992	0.955	0.1057	89.43
P	0.058	0.058	1.149	0.361	0.3959	60.41
Residual	0.100					
Cor Total	86.272					
Std. Dev.		0.2238		R-Squared		0.9988
Mean		2.3269		Adj R-Squared		0.9890
C.V. %		9.6187		Pred R-Squared		0.8839
PRESS		10.0187		Adeq Precision		32.5110

*SS* - sum of squares, *MS*- mean square, *F*: Fishers’s function, *P*: Level of significance, *PRESS* the predicted residual sum of squares, CV %-the coefficient of variation%

*Significant values

The Pareto chart illustrates the order of significant effects of the variables affecting cholesterol oxidase production in Plackett-Burman experimental design (Fig. 4b). It displays the absolute values of the effects, and draws a reference line on the chart. Any effect that extends past this reference line is potentially important.

The percentages of contributions of the variables are given in Table 5. The results revealed that inoculum size, (NH_4_)_2_SO_4_, pH and cholesterol are the most contributing variables with 15.323, 13.909, 12.521 and 12.293 %; respectively.

The analysis of variance (ANOVA) of the experimental design was calculated, and the sum of square, mean square, *F*-value, *t*-value, *P*-value and confidence level are given in Table 6. The significance of the model was calculated by the *P*-value. The *P*-value (probability value) serves as a tool for checking the significance of each of the parameter. The model *F*-value of 101.19 and *P*-value of 0.0098 implies that the model is significant. There is only a 0.98 % chance that a “Model *F*-Value” this large could occur due to noise. Values of “Prob > *F*” (*P*-value) less than 0.05 indicate model terms are significant.

The data revealed that, nine variables (temperature, incubation time, inoculum size, pH, glucose, cholesterol, yeast extract, peptone and (NH_4_)_2_SO_4_) were found to significantly affect cholesterol oxidase production while the remaining six variables (agitation speed, starch, K_2_HPO_4_, NaCl, MgSO_4_.7H_2_O and FeSO_4_.7H_2_O) have not significant effect on the cholesterol oxidase production (Table 6). In this connection the analysis showed that, inoculum size (C) with a probability value of 0.0026 was determined to be the most significant factor affecting cholesterol oxidase production by *Streptomyces cavourensis* strain NEAE-42 at 99.74 % confidence followed by (NH_4_)_2_SO_4_ (L) (*P*-value = 0.0031), pH (E) (*P*-value = 0.0039) and cholesterol (H) (*P*-value = 0.004), the lower probability values indicate the more significant variables affecting cholesterol oxidase production. Also, it was clear that among the four variables, only inoculum size exerted a negative effects, whereas the other variables (pH, (NH_4_)_2_SO_4_ and cholesterol) exerted positive effect on cholesterol oxidase production, which means that the increase in the concentrations of pH value, (NH_4_)_2_SO_4_ and cholesterol concentration and decrease in inoculum size could exert positive effect on cholesterol oxidase production.

The R^2^ values provide a measure of how much variability in the observed response values can be explained by the experimental variables. The R^2^ value is always between 0 and 1. When R^2^ is closer to the 1, the model is stronger and better to predict the response [36]. The value of the determination coefficient (R^2^) was found to be 0.9988 indicates that 99.88 % of the variability in cholesterol oxidase production could be explained by the independent variables used in the study and only 0.12 % of the total variations are not explained by these variables. The adjusted R^2^ (0.9890) is also very high that indicates that the model is very significant [37]. The “Pred R-Squared” of 0.8839 is in reasonable agreement with the “Adj R-Squared” of 0.9890. This indicated a good adjustment between the experimental and predicted values. “Adeq Precision” measures the signal to noise ratio. A ratio greater than 4 is desirable. Our ratio of 32.511 indicates an adequate signal.

A lower value of the coefficient of variation % (CV % = 9.6187 %) indicates a greater reliability of the experimental performance. The predicted residual sum of squares (PRESS) is a measure of how well the model fits each point in the design. The smaller the PRESS statistic, the better the model fits the data points. Our value of PRESS is 10.0187. The model shows standard deviation and mean value of 0.2238 and 2.3269, respectively.

The first order polynomial equation was derived after performing regression analysis on the results and representing cholesterol oxidase production as a function of the independent variables to obtain the optimum response. By neglecting the insignificant variables, the following regression equation in terms of coded variables was obtained**:**

3 $$ \begin{array}{l}{\mathrm{Y}}_{\left(\mathrm{Cholesterol}\ \mathrm{oxidase}\ \mathrm{activity}\right)}=+2.33\ \hbox{-} 0.26\mathrm{A}+0.42\ \mathrm{B}\hbox{-} 0.98\mathrm{C}+0.18\mathrm{D}+0.80\mathrm{E}\hbox{-} 0.42\mathrm{F}+0.13\mathrm{G}+0.79\mathrm{H}\hbox{-} \\ {}0.31\mathrm{J}+0.76\mathrm{K}+0.89\mathrm{L}+0.064\mathrm{M}\hbox{-} 0.21\mathrm{N}+0.14\mathrm{O}+0.054\ \mathrm{P}\hbox{-} 0.18\mathrm{S}\hbox{-} 0.22\mathrm{T}\end{array} $$

Where Y is cholesterol oxidase production, and A, B, C, E, F, H, J, K and L are temperature, incubation time, inoculum size, pH, glucose, cholesterol, yeast extract, peptone and (NH_4_)_2_SO_4_ respectively. The coefficient of each variable represents the effect extent of these variables on cholesterol oxidase production.

On the basis of *t* Stat (Table 6), cholesterol concentration, pH and (NH_4_)_2_SO_4_ were chosen for further optimization using central composite design experiments (CCD), since these factors had the most positive significant effects on cholesterol oxidase production.

### Model adequacy checking

**Fig. 5:**
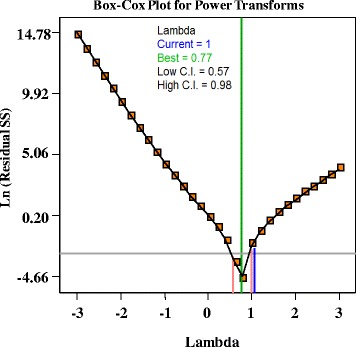
Box- Cox plot of model transformations

As observed from Box–Cox plot (Fig. 5), the blue line indicates the current transformation (= 0.77) and the green line indicates the best lambda value (= 0.77), while the red lines indicate the minimum and maximum 95 % confidence interval values (0.57 and 0.98 respectively). So that the model is well fit to the experimental data obtained and well satisfies the assumptions of the analysis of variance.

In a confirmatory experiment, to evaluate the accuracy of Plackett-Burman, a medium of the following composition: Temperature 30 °C; incubation time 7 days; inoculum size 2(%, v/v); agitation speed 200 rpm; pH 8; glucose 10 g/L; starch 10 g/L; cholesterol 2 g/L; yeast extract 4 g/L; peptone 5 g/L; (NH_4_)_2_SO_4_ 8 g/L; K_2_HPO_4_ 1 g/L; NaCl 0.5 g/L; MgSO_4_.7H_2_O 0.5 g/L; FeSO_4_.7H_2_O 0.02 g/L, which expected to be optimum gives cholesterol oxidase activity of 6.732 U/mL which is higher than result obtained from the basal medium before applying Plackett Burman by more than two times (3.31 U/mL).

Various compounds, such as cholesterol, yeast extract [10], yeast extract, potato starch, peptone and malt extract [38] have been recorded to be substrates for an enhanced cholesterol oxidase production. Cholesterol is utilized widely by different microorganisms as a carbon and energy source [12]. Different microorganisms such as *Brevibacterium, Corynebacterium, Arthrobacter, Nocardia, Mycobacterium,* and *Streptomyces* have the ability to degrade cholesterol. The first step of microbial assimilation of cholesterol is the oxidation of the 3β- hydroxyl group by cholesterol oxidase [39]. Therefore, cholesterol assimilating microorganisms are generally considered to produce cholesterol oxidases. Yehia et al. [40] reported that the growth and assimilation of cholesterol by the tested bacterial isolates were affected greatly by concentration of cholesterol used in the fermentation medium, whereas the maximum percentage of cholesterol assimilation (80.2 %) by the *Enterococcus hirae* strain was achieved at 1 g/L of the added cholesterol and maximal assimilation of cholesterol by *Streptomyces fradiae* [10] and *Rhodococcus erythropolis* ATCC 25544 [41] was obtained when used 2 g/L.

The pH of the cultivation medium is very important for the microbial growth and metabolism, and hence, for the production of metabolites. The pH may have a direct effect on the cell, or it may indirectly affect it by varying the dissociation degree of the medium components [42]. The pH value of the culture medium plays a critical role in the optimal physiological performance of the cells and the transport of various nutrient components across the cell membrane, and the cholesterol assimilation is affected by a change in the pH value of the media. It was previously reported that the optimal pH values for cholesterol assimilation are 7.2 for *Streptomyces fradiae* [10] and 6.75 for *Rhodococcus erythropolis* ATCC 25544 [41]. Moreover, it was found that, optimal pH value required to attain maximum growth and assimilation of cholesterol (80.2 %) in the liquid medium by *Enterococcus hirae* was pH 7.0 using 0.2 M acetate buffer and 0.2 M phosphate buffer [40]. Solingen et al. [43] reported that an alkaline novel *Streptomyces* species isolated from east african soda lakes have an optimal pH 8.

Voelker and Altaba [44] estimated the role of different organic and inorganic nitrogen sources for growth and production of secondary metabolite from a *Streptomycete*s. In general, cholesterol oxidase production was enhanced far more by using organic nitrogen than inorganic nitrogen. This may be due to organic nitrogen contains most types of growth factors and amino acids important for the bacterial growth and could be metabolized by cells directly, consequently enhancing cholesterol oxidase production [45]. Sabry [46] reported that ammonium sulphate, sodium nitrate and ammonium nitrate are the best nitrogen sources used for cholesterol assimilation by *Pseudonocardia compacta* S-39. Ammonium salts have shown the highest effect on cholesterol oxidase production by *Arthrobacter simplex* [47]. The cell mass obtained using the growth stimulating nitrogen sources, namely yeast extract and (NH_4_)_2_HPO_4_, supports the cholesterol oxidase accumulation as induced by the cholesterol substrate in the cell wall [48]. Among inorganic and organic nitrogen sources, it was revealed that yeast extract had more influence on cholesterol oxidase production than (NH_4_)_2_SO_4_ (inorganic nitrogen source) [49]. According to Lee et al. [50], study on *Rhodococcus equi* no. 23 showed that yeast extract at 0.4–0.5 % w/v was the best nitrogen source for cholesterol oxidase production. In a similar study, *Rhodococcus equi* 2C showed maximum production of cholesterol oxidase with yeast extract at 0.3 %w/v [10].

### Statistical optimization of fermentation process variables for cholesterol oxidase production using central composite design (CCD)

The results indicate the effectiveness of the Plackett-Burman design in identifying the factors with a positive significant influence on the cholesterol oxidase production. As Plackett-Burman design is inappropriate to study the mutual interaction of process variables, therefore the level of significant factors needed further optimization. Thereafter the exact optimal values for the individual significant factors were determined using central composite design experiments. The significant variables with positive effect were fixed at high level. The variables which exerted a negative effect on cholesterol oxidase production were maintained in all trials at their low level for further optimization by central composite design; other insignificant variables were set at their low level of Placket-Burman design design as the following: Temperature 30 °C; incubation time 7 days; inoculum size 2 (%, v/v); agitation speed 150 rpm; glucose 10 g/L; starch 7 g/L; yeast extract 4 g/L; peptone 5 g/L; K_2_HPO_4_ 0.5 g/L; NaCl 0.5 g/L; MgSO_4_.7H_2_O 0.2 g/L.

Placket-Burman design results revealed that, pH (X_1_), cholesterol concentration (X_2_), and (NH_4_)_2_SO_4_ (X_3_) were the most significant positive independent variables affecting cholesterol oxidase production, thus they were selected for further optimization using five level central composite design (CCD). Table 7 shows the three independent variables and their concentrations at different coded and actual levels of the variables employed in the design matrix.

**Table 7:** Central composite design representing the response of cholesterol oxidase production by *Streptomyces cavourensis* strain NEAE-42 as influenced by initial pH (X_1_), cholesterol (X_2_) and ammonium sulphate (X_3_) along with the predicted cholesterol oxidase production and residuals and the levels of variables with actual factor levels corresponding to coded factor levels

Std	Run	Type	Variables			Cholesterol oxidase activity (U/mL)		Residuals
			X_1_	X_2_	X_3_	Experimental	Predicted	
14	1	Axial	0	0	1.68	6.362	5.970	0.392
11	2	Axial	0	−1.68	0	18.184	17.893	0.290
7	3	Factorial	−1	1	1	10.612	10.674	−0.062
3	4	Factorial	−1	1	−1	16.506	15.551	0.955
4	5	Factorial	1	1	−1	15.367	14.300	1.067
19	6	Center	0	0	0	20.403	20.365	0.038
5	7	Factorial	−1	−1	1	13.655	14.060	−0.405
2	8	Factorial	1	−1	−1	13.630	12.906	0.724
12	9	Axial	0	1.68	0	14.992	16.218	−1.226
9	10	Axial	−1.68	0	0	15.492	15.476	0.016
10	11	Axial	1.68	0	0	11.401	12.353	−0.951
1	12	Factorial	−1	−1	−1	14.431	14.408	0.023
6	13	Factorial	1	−1	1	11.303	11.596	−0.293
17	14	Center	0	0	0	20.348	20.365	−0.017
18	15	Center	0	0	0	20.424	20.365	0.059
20	16	Center	0	0	0	20.279	20.365	−0.086
16	17	Center	0	0	0	20.521	20.365	0.156
13	18	Axial	0	0	−1.68	9.845	11.172	−1.327
8	19	Factorial	1	1	1	9.101	8.462	0.639
15	20	Center	0	0	0	20.376	20.365	0.011
Level		pH	Cholesterol (g/L)			Ammonium sulphate (g/L)		
−1.68		6	1			5		
−1		7	2			6		
0		8	3			8		
1		9	4			9		
1.68		10	5			10		

Central composite design matrix and responses (experimental and predicted cholesterol oxidase) for the 20 runs of the design are presented in Table 7, which shows considerable variation in the amount of cholesterol oxidase activity. Based on the experimental data obtained; cholesterol oxidase activity ranged from 6.362 to 20.521 U/mL, the highest levels of cholesterol oxidase activity were obtained in runs 6, 14, 15, 16, 17 and 20 (center points) with values of 20.403, 20.348, 20.424, 20.279, 20.521 and 20.376 U/mL respectively, where pH 8, cholesterol concentration 3 g/L, (NH_4_)_2_SO_4_ 8 g/L were used, while the minimum cholesterol oxidase activity was observed in run number 1 where pH 8, cholesterol concentration 3 g/L, (NH_4_)_2_SO_4_ 10 g/L were used. In Table 7, the observed values for cholesterol oxidase activity is compared with the predicted values from the model.

The data were analyzed using Design Expert® 7.0 for Windows to perform statistical analysis. The determination coefficient (R^2^) of the model was 0*.*9794 (Table 8) indicating that 97.94 % of variability in the production of cholesterol oxidase was attributed to the selected independent variables and only 2.06 % % of the total variance could not be explained by the model. The highest R^2^ value showed the good agreement between the experimental results and the predicted values by the model [51]. If R^2^-value of the regression model is higher than 0.9, it was considered as having a very high correlation [52]. Therefore, the present R^2^-value reflected a very good fit between the observed and predicted responses, and implied that the model is reliable for cholesterol oxidase production in the current study. The “Pred R-Squared” of 0.8434 is in reasonable agreement with the “Adj R-Squared” of 0.9608. This indicated a good correlation between the predicted and observed values. “Adeq Precision” measures the signal to noise ratio. A ratio greater than 4 is desirable. Our ratio of 23.224 indicates an adequate signal. Usually, the higher value of the coefficient of variation % (CV %) indicated the lower reliability of the experiment, in the present study, a lower value of C.V. (5.7815) indicated a better reliability of the experimental results [53]. The predicted residual sum of squares (PRESS) is a measure of how well the model fits each point in the design. The smaller the PRESS statistic indicates better model fits for the data points. Our value of PRESS is 58.33. The model shows standard deviation and mean value of 0.876 and 15.16, respectively (Table 8). The positive coefficients for X_1_X_2_ (Table 8) indicate that the interaction effect between the two variables increase cholesterol oxidase production, while negative coefficients indicate that the interaction effect between the two variables decrease in cholesterol oxidase production.

**Table 8:** Regression statistics of CCD for optimization of cholesterol oxidase production by *Streptomyces cavourensis* strain NEAE-42

Factor	Coefficient estimate	Standard error	95 % CI Low	95 % CI High
Intercept	20.365	0.358	19.569	21.162
X_1_ (initial pH)	−0.929	0.237	−1.457	−0.400
X_2_ (cholesterol)	−0.498	0.237	−1.026	0.031
X_3_ (ammonium sulphate)	−1.547	0.237	−2.075	−1.018
X_1_ X_2_	0.063	0.310	−0.628	0.753
X_1_ X_3_	−0.240	0.310	−0.931	0.450
X_2_ X_3_	−1.132	0.310	−1.823	−0.442
X_1_^2^	−2.281	0.231	−2.795	−1.766
X_2_^2^	−1.170	0.231	−1.685	−0.656
X_3_^2^	−4.170	0.231	−4.684	−3.655
Std. Dev.	0.8766	R-Squared		0.9794
Mean	15.1615	Adj R-Squared		0.9608
C.V. %	5.7815	Pred R-Squared		0.8434
PRESS	58.3337	Adeq Precision		23.2242
C.V: Coefficient of variation				

The model adequacy was checked using analysis of variance (ANOVA) which was tested using Fisher’s statistical analysis and the results are shown in Table 9. The Model *F*-value of 52.77 indicate the model is significant. There is only a 0.01 % chance that a “Model *F*-Value” this large could occur due to noise. Values of “Prob > *F*” less than 0.05 indicate model terms are significant. In this case X_1_, X_3_, X_2_ X_3_, X_1_^2^, X_2_^2^, X_3_^2^ are significant model terms. Values greater than 0.1 indicate the model terms are not significant. The “Lack of Fit *F*-value” of 233.149 implies the Lack of Fit is significant. There is only a 0.01 % chance that a “Lack of Fit *F*-value” this large could occur due to noise (Table 9).

**Table 9:** Analysis of variance (ANOVA) for CCD results used for optimizing cholesterol oxidase production by *Streptomyces cavourensis* strain NEAE-42

Source	Sum of Squares	*df*	Mean Square	*F-*value	*P-*value *P*rob > *F*
Model	364.929	9	40.548	52.771	<0.0001*
X_1_ (initial pH)	11.778	1	11.778	15.328	0.0029*
X_2_ (cholesterol)	3.386	1	3.386	4.406	0.0622
X_3_ (ammonium sulphate)	32.666	1	32.666	42.513	<0.0001*
X_1_ X_2_	0.032	1	0.032	0.041	0.8432
X_1_ X_3_	0.462	1	0.462	0.602	0.4558
X_2_ X_3_	10.254	1	10.254	13.344	0.0044*
X_1_^2^	74.961	1	74.961	97.557	<0.0001*
X_2_^2^	19.730	1	19.730	25.677	0.0005*
X_3_^2^	250.574	1	250.574	326.109	<0.0001*
Residual	7.684	10	0.768		
Lack of Fit	7.651	5	1.530	233.149	<0.0001*
Pure Error	0.033	5	0.007		
Cor Total	372.613	19			

*df* : Degree of freedom, *F*: Fishers’s function, *P* : Level of significance

*Significant values

The fit summary results are presented in Table 10, contributed to find an adequate type of response surface model. Quadratic model type was selected to be the proper model that fit the CCD of cholesterol oxidase production by *Streptomyces cavourensis* strain NEAE-42, where the results of fit summary showed that, the quadratic model is a highly significant with a very low probability value [(*P*_model_ > *F*) < 0.0001]. The model summary statistics of the quadratic model showed the smallest standard deviation of 0.877 and the largest predicted and adjusted R-squared of 0.961 and 0.843 respectively.

**Table 10:** Fit summary for experimental data

Sequential Model Sum of Squares					
Source	Sum of Squares	*df*	Mean Square	*F-*value	*P-*value (Prob > *F*)
Linear vs Mean	47.829	3	15.943	0.785	0.5194
Two factors interaction (2FI) vs Linear	10.748	3	3.583	0.148	0.9289
Quadratic vs 2FI	306.352	3	102.117	132.900	<0.0001*
Residual	2.226	6	0.371		
Lack of Fit Tests					
Source	Sum of Squares	*df*	Mean Square	*F-*value	*P-*value (Prob > *F*)
Linear	324.750	11	29.523	4498.259	<0.0001*
Two factors interaction (2FI)	314.003	8	39.250	5980.409	<0.0001*
Quadratic	7.651	5	1.530	233.149	<0.0001*
Pure Error	0.033	5	0.007		
Model Summary Statistics					
Source	Standard deviation	R-Squared	Adjusted R-Squared	Predicted R-Squared	PRESS
Linear	4.505	0.128	−0.035	−0.277	475.939
Two factors interaction (2FI)	4.915	0.157	−0.232	−0.826	680.540
Quadratic	0.877	0.979	0.961	0.843	58.334

*df* : degree of freedom, PRESS: sum of squares of prediction error

* Significant values

A second-order polynomial model (Eq. 4) was proposed to calculate the optimum levels of pH, cholesterol concentration and (NH_4_)_2_SO_4_, to evaluate the relationship between cholesterol oxidase production and independent variables and to determine the maximum cholesterol oxidase production corresponding to the optimum levels of these variables. The second-order polynomial equation that defines predicted response (Y) in terms of the independent variables (X_1_, X_2_ and X_3_) was obtained:

4 $$ \begin{array}{l}{{\mathrm{Y}}_{\Big(}}_{\mathrm{Cholesterol}\ \mathrm{oxidase}\ \mathrm{activity}\Big)} = +20.37-0.93{\mathrm{X}}_1-0.50{\mathrm{X}}_2-1.55{\mathrm{X}}^3+0.063{\mathrm{X}}_1{\mathrm{X}}_2-0.24{\mathrm{X}}_1{\mathrm{X}}_3-1.13{\mathrm{X}}_2{\mathrm{X}}_3-2.28\;\\ {}{{\mathrm{X}}_1}^2-1.17{{\mathrm{X}}_2}^2-4.17{{\mathrm{X}}_3}^2\end{array} $$

Where the Y is the predicted cholesterol oxidase activity, pH (X_1_), cholesterol concentration (X_2_), and (NH_4_)_2_SO_4_ (X_3_).

### Three dimensional plots

**Fig. 6:**
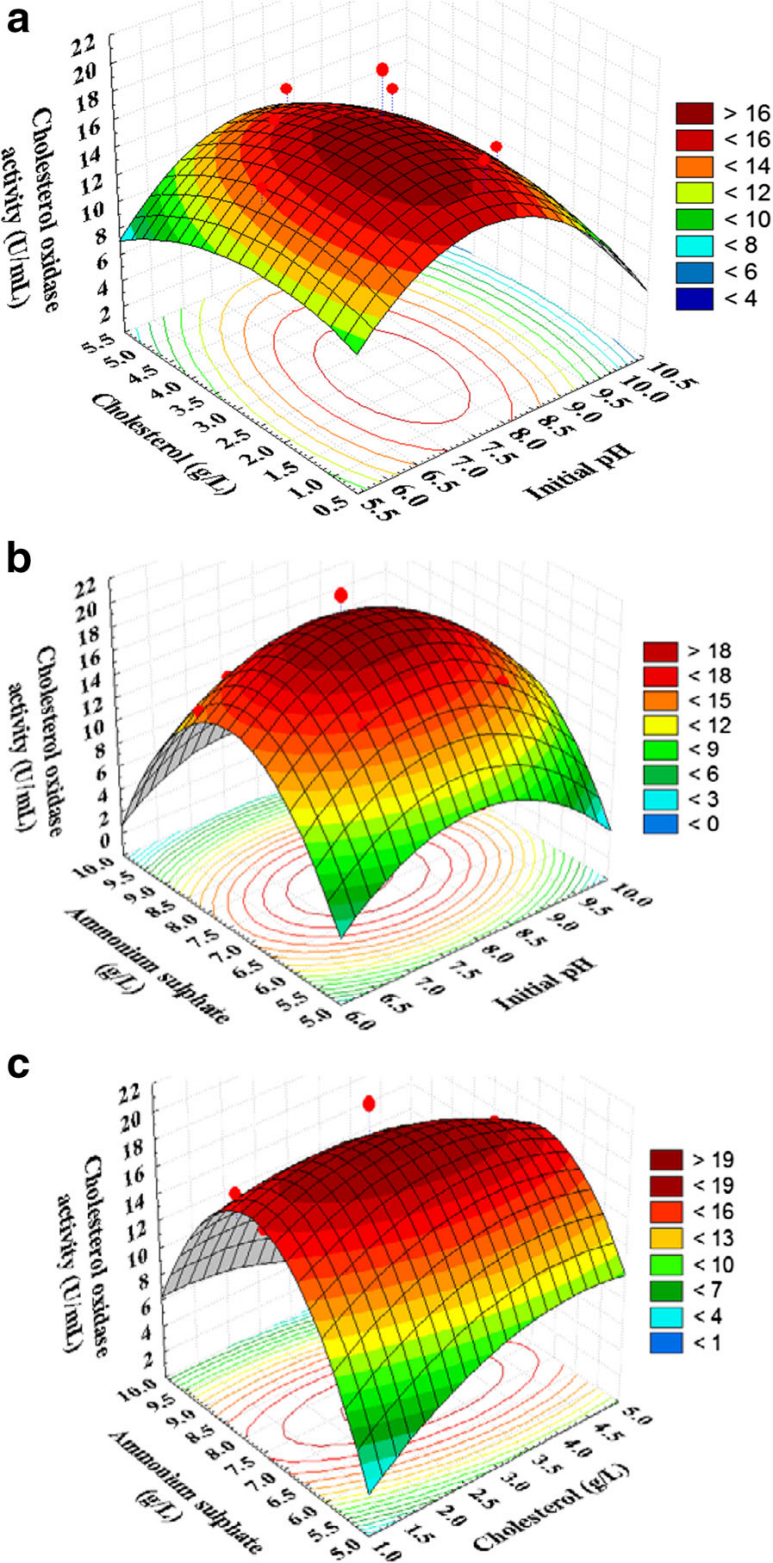
Three-dimensional response surface plots initial pH (X_1_), cholesterol (X_2_) and ammonium sulphate (X_3_) on the cholesterol oxidase activity

The three dimensional response surface curves were plotted to understand the interaction of the variables and the optimal levels of each variable required for the optimal cholesterol oxidase production. Three dimensional plots for the combinations of the three variables (X_1_ X_2_, X_1_ X_3,_ and X_2_ X_3_) were generated by plotting the response (cholesterol oxidase production) on Z-axis against two independent variables while keeping the other variable at its center point (shown in Figs. 6a–c).

The 3D plot (Fig. 6a), showing the effects of initial pH (X_1_), and cholesterol (X _2_) on cholesterol oxidase production. It can be seen that, when the initial pH increases, cholesterol oxidase production gradually increases, but further increase in initial pH above 7.5 leads to decrease in cholesterol oxidase production. It showed that lower and higher levels of cholesterol support relatively low levels of cholesterol oxidase activity; cholesterol oxidase activity was increased with increase in cholesterol concentration. The maximum cholesterol oxidase activity was attained at middle levels of cholesterol. Figure 6b represents the cholesterol oxidase activity as a function of initial pH (X_1_), ammonium sulphate (X_3_) by keeping cholesterol (X_2_) at optimum value, the maximum cholesterol oxidase activity was attained at moderate levels of both initial pH and ammonium sulphate and further increase resulted in a gradual decrease in the cholesterol oxidase activity. In addition, the interaction between these variables were not significant, indicating that there is no significant correlation between each two variables and that they did not help much in increasing the production of cholesterol oxidase production. Figure 6c represents the cholesterol oxidase activity as a function of cholesterol (X_2_), ammonium sulphate (X_3_) by keeping initial pH (X_1_) at optimum value, the maximum cholesterol oxidase activity was attained at moderate levels of ammonium sulphate and further increase resulted in a gradual decrease in the cholesterol oxidase activity. It was observed that there is increasing in cholesterol oxidase activity with increasing cholesterol concentration, the maximum cholesterol oxidase activity was obtained at moderate levels and further increase resulted in a gradual decrease in the cholesterol oxidase activity. In addition, the interaction between these variables were significant, indicating that there is significant correlation between each two variables and that they help much in increasing the production of cholesterol oxidase activity.

### Model adequacy checking

**Fig. 7:**
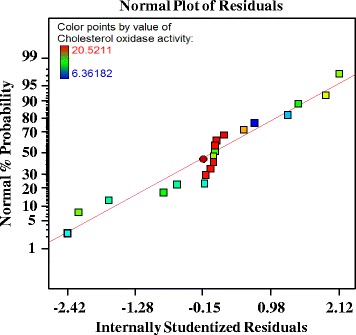
The normal probability plot of residuals to check for normality of residuals for cholesterol oxidase production by *Streptomyces cavourensis* strain NEAE-42 determined by the second-order polynomial equation

The normal probability plot is an important diagnostic tool which indicates whether the residuals follow a normality assumption, in which case the points will follow a straight line expect some scatter even with normal data. Figure 7 showed that, the normality assumption was satisfied as the residual plot approximated a long a straight line for cholesterol oxidase production, this indicates that the model was well fitted with the experimental results. As the residuals from the fitted model were normally distributed, all the major assumptions of the model had been validated.

### Validation of the model

The model was validated by repeating the experiments under the optimized conditions, which resulted in the cholesterol oxidase production of 20.279 U/mL (predicted response 20.365 U/mL), indicating a strong agreement between them and proving the validity of the model.

## Conclusion

Little data has been reported in the literature on the optimization of cholesterol oxidase production by microorganisms using statistical methods. The level of cholesterol oxidase production obtained in our study (20.521U/mL) following the statistical method is higher than many of the reported values [10, 38, 45].

## Abbreviations

ANOVA: The analysis of variance
CCD: Central composite design
CV: The coefficient of variation%
HDL: High-density lipoprotein
HPLC: High performance liquid chromatography
LDL: Low-density lipoprotein
PB: Plackett-Burman
PCR: The polymerase chain reaction
PRESS: The predicted residual sum of squares
