# Erratum to: Identification and statistical optimization of fermentation conditions for a newly isolated extracellular cholesterol oxidase producing *Streptomyces cavourensis* strain NEAE-42

**DOI:** 10.1186/s12866-016-0875-4

**Published:** 2016-11-04

**Authors:** Noura El-Ahmady El-Naggar, Nancy M. El-Shweihy, Sara M. El-Ewasy

**Affiliations:** Department of Bioprocess Development, Genetic Engineering and Biotechnology Research Institute, City of Scientific Research and Technological Applications, New Borg El-Arab City, Alexandria 21934 Egypt

## Update and Erratum

Unfortunately, a number of corrections had not been carried out during the production of the original version of this article [1].

Within the paragraph headed ‘Chemotaxonomy and physiological characteristics’ the reference to Fig. 2b has been corrected to Fig. 1b.

*Streptomyces flavolimosus* has been italicised.

The sentence ‘As observed from Box–Cox plot (Fig. 5), the blue line indicates the current transformation (=0.77)’ has been changed to ‘As observed from Box–Cox plot (Fig. 5), the blue line indicates the current transformation (Lambda = 1)’.

Table 7 has been clearly formatted to include lines.

Numbers in the equation for Y (Cholesterol oxidase activity) have been corrected to subscript:

$$ {{\mathbf{Y}}_{\mathbf{\Big(}}}_{\mathbf{Cholesterol}\ \mathbf{oxidase}\ \mathbf{activity}\mathbf{\Big)}}=+20.37-0.93\;{\mathrm{X}}_1-0.50\;{\mathrm{X}}_2-1.55\;{\mathrm{X}}_3+0.063\;{\mathrm{X}}_1{\mathrm{X}}_2-0.24\;{\mathrm{X}}_1{\mathrm{X}}_3-1.13\;{\mathrm{X}}_2{\mathrm{X}}_3-2.28\;{{\mathrm{X}}_1}^2-1.17\;{{\mathrm{X}}_2}^2-4.17\;{{\mathrm{X}}_3}^2 $$

The caption for Fig. 6 has been amended to ‘Three-dimensional response surface plots for the effect of initial pH (X_1_), cholesterol (X_2_) and ammonium sulphate (X_3_) on the cholesterol oxidase activity’.
